# Posterior hamstring harvest improves aesthetic satisfaction and decreases sensory complications as compared to the classic anterior approach in anterior cruciate ligament reconstruction surgery

**DOI:** 10.1186/s40634-022-00547-y

**Published:** 2022-11-03

**Authors:** Jesús Manuel García Hernández, Emilio López-Vidriero Tejedor, Sofía Castañeda González, Joaquín Yrayzoz Fuentes, Rafael Periáñez Moreno, Jose María Saval Benítez, Guillermo Carrascal Aldana

**Affiliations:** 1 Orthopaedic Surgery and Traumatology Department, Hospital Universitario Virgen Macarena, Seville, Spain; 2 Orthopaedic and Arthroscopic Knee Surgery Unit, Hospital Universitario Virgen Macarena, Seville, Spain; 3 Head of Orthopaedic and Arthroscopic Knee Surgery Unit, Hospital Universitario Virgen Macarena, Seville, Spain; 4 Head of Orthopaedic Surgery and Traumatology Department, Hospital Universitario Virgen Macarena, Seville, Spain

**Keywords:** ACL reconstruction, Semitendinosus tendon, Saphenous nerve injury, Popliteal fossa, Posterior harvesting

## Abstract

**Purpose:**

The use of the posterior approach for harvesting hamstring grafts has recently become popular thanks to new all-inside techniques and retrograde drills. This study aims to compare the classic anterior approach with the posterior approach in the popliteal fossa.

**Methods:**

Retrospective comparative study of 100 consecutive cases of primary ligamentoplasty performed using ipsilateral semitendinosus autograft with at least one year of follow-up. 50 patients with anterior approach (group A) and 50 patients with posterior approach (P). Ratio men/women: 9/1. Mean age: 32 ± 13 years. Mean operative time: 64.88 ± 12.28 min.

**Study variables:**

Graft harvest time; intraoperative complications (semitendinous [ST] tendon cut); postoperative neurological complications (allodynia, paresthesia, pain) or hematoma in the donor area; atrophy of the operated thigh compared to the contralateral thigh, postoperative VAS score, aesthetic satisfaction and overall satisfaction.

**Results:**

Graft harvest time of 9.5 min in group A versus 5.25 min in group P (*p* < 0.05). Sensory complications: 16% in group A versus 2% in group P (*p* < 0.05). Regarding the patient’s evaluation of the aesthetic result of the surgery, 80% in group A and 92% in group P were very satisfied, 16% in group A and 8% in group P were satisfied and 4% in group A and no patients in group P not very satisfied (*p* < 0.05). No significant differences were found in terms of total operative time, postoperative joint movement, atrophy of the operated thigh, postoperative VAS, or overall patient satisfaction.

**Conclusions:**

The posterior approach to harvesting the ipsilateral hamstring graft obtained better results than the anterior approach in terms of aesthetic satisfaction of the patient, lower rate of neurological complications (allodynia, paresthesias and hypoesthesia in the anterior region of the knee and leg) and shorter hamstring harvest time.

**Level of evidence:**

IV.

## Introduction

Anterior cruciate ligament reconstruction (ACLR) is one of the most common surgeries in sports traumatology [13]. In the United States, there are more than 200,000 injuries each year, resulting in an estimated total cost of approximately $7 billion between direct and indirect costs [11, 16, 24]. With the increase in sport activity worldwide, the number of ACL injuries has also increased, both in adults and in adolescents [6, 7, 9, 11, 12, 23, 27, 30]. As a result, increasingly more research is being conducted on this pathology and every aspect of ACL surgery.

There are various types of grafts in the repertoire of sports knee surgeons, including those harvested from the hamstring, patellar tendon, and quadriceps tendon, as well as several types of allografts [3, 8, 14]. The ACL Study Group recently conducted an updated survey of international surgeons who perform high volume ACLR. It was found that the type of graft most widely used in primary ACLR was the semitendinosus autograft (53%), sometimes with the support of the gracilis tendon. This was followed by the use of the bone-patellar tendon-bone (BPTB) graft (36%), the quadriceps tendon (QT), allografts, and other options [24]. When asked which choice of graft they believed would be used most commonly 10 years from now, participating surgeons predicted that the hamstrings would continue to be predominant, most likely due the lower rate of complications associated with the donor area, among other reasons [15, 24, 29].

There are various cutaneous (vertical, horizontal, oblique) and localization (anterior and posterior) approaches available to harvest the hamstring graft and make the operation quicker and easier for the surgeon, while limiting complications and improving satisfaction for the patient. Traditionally, an anterior approach has used in the anteromedial pretibial area, taking advantage of the fact that the tibial tunnel is drilled anterograde and interference fixation is placed in the same area. One disadvantage of the anterior approach is that it can damage the infrapatellar and/or sartorius branch of the saphenous nerve. Moreover, because the pes anserinus is formed by three distally conjoined tendons, with the sartorius muscle so close by, it can be more complicated to separate the semitendinosus and gracilis tendons for harvesting. Furthermore, the medial collateral ligament lies deep to the tendons, which can cause confusion and is sometimes extracted iatrogenically as a graft [17, 22].

More recently, thanks to the use of retrograde drills to make blind tunnels while preserving the bone stock and to avoid the issues associated with the anteromedial approach, the posterior approach in the posteromedial aspect of the popliteal fossa has become popular for graft harvesting, as published by Kodkani [10] in 2004 and later by Prodromos [19] in 2005 and Franz in 2016 [5]. The aim of this approach is to reduce the theoretical drawbacks of anterior approaches by avoiding the infrapatellar and/or sartorial branch of the saphenous nerve. Furthermore, both tendons are individualized at the subcutaneous level, making them easier to locate and preventing confusion with the medial collateral ligament.

Interestingly, an exhaustive review of the bibliography yielded just one published article comparing these two approaches in terms of operative time and neurological complications, concluding that the posterior approach is quicker and results in a lower rate of sensory disturbances [5]. There is an additional study that compares the approaches to assess the recovery of the hamstrings and quadriceps, finding no differences for the hamstrings and better quadricep strength after three months of the posterior approach [4].

The aim of this study was to compare these hamstring harvesting approaches based on the hypothesis that the posterior approach is quicker, results in fewer complications and leads to greater aesthetic satisfaction [20, 21, 28].

## Materials and methods

### Design and study cohort

Retrospective comparative study of consecutive cases of primary ACLR performed using ipsilateral hamstring autograft, with and without associated meniscal suture. The analysis performed was a comparative study of the group of patients who underwent an anterior approach (group A) and those who underwent a posterior approach (group P) for the graft harvest. The patients were operated on between March 2017 and March 2020 by two surgeons from the hospital's Knee Unit, both with more than 10 years of experience in knee surgery. One of them performed surgery by harvesting the graft via the anterior approach in group A with dynamic femoral cortical suspension and interference screw in the tibia; the other used a posterior approach in group P with an “all-inside” technique and dynamic suspension in the femur and tibia.

The inclusion criteria were postoperative follow-up of more than a year, legal age and complete ACL tear with or without associated meniscal injury. The exclusion criteria were multiligament injuries and associated secondary stabilization and revision surgeries.

### Surgical technique

**Fig. 1:**
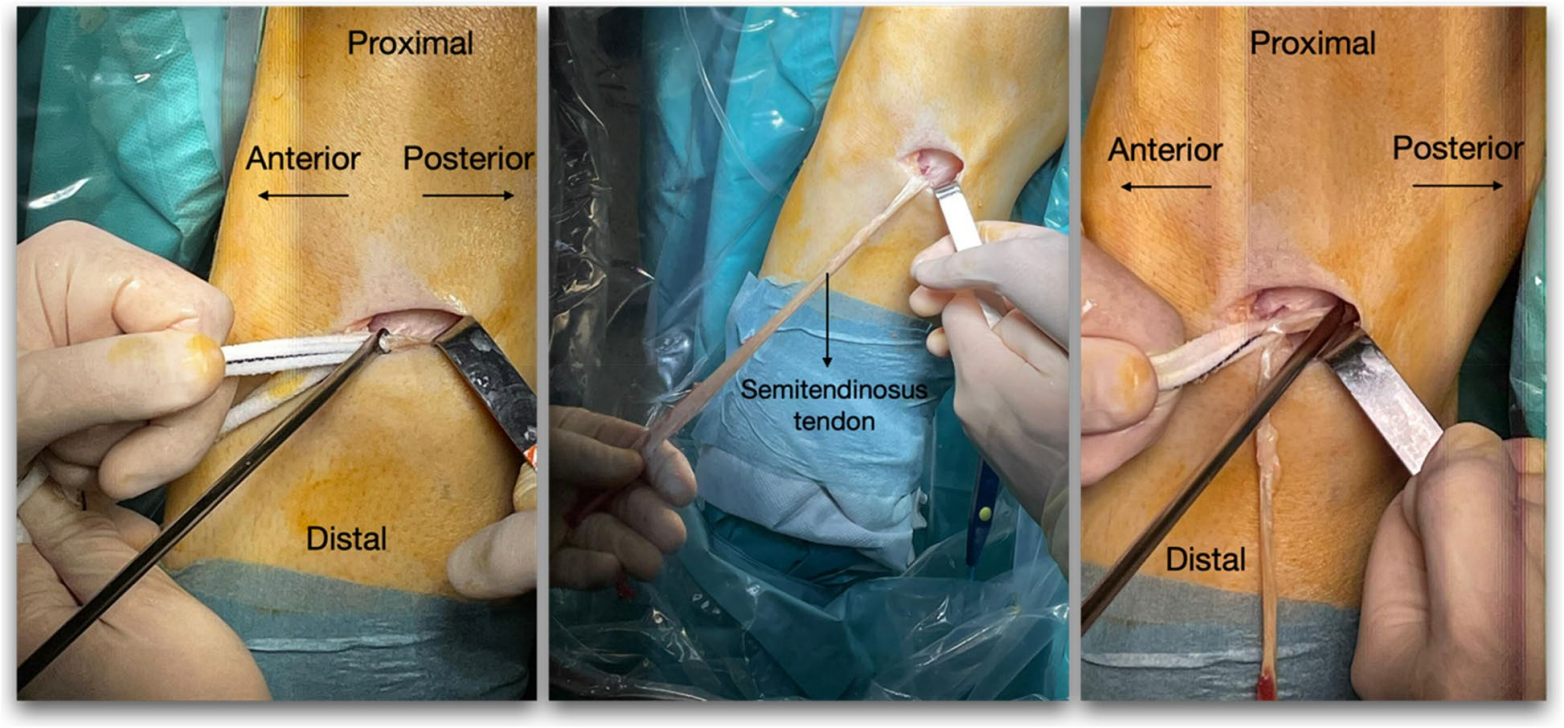
Anterior harvesting technique; Left: Insertion of a tenotomy knife to release the semitendinosus muscle tendon. Middle: Identification of the gracilis muscle tendon. Right: Insertion of a closed tenotome knife to release the gracilis muscle tendon

In group A, the hamstring graft was harvested by means of an anterior approach, with a 4–5 cm ascending oblique incision using the lateral margin of the anterior tibial tuberosity as a reference. The fascia of the sartorius muscle and the joint semitendinosus and gracilis tendon were then opened, after which both tendons were disinserted and the bands of the medial gastrocnemius were released. Lastly, the grafts were extracted from their muscular insertion with a closed tenotomy (Fig. 1).

**Fig. 2:**
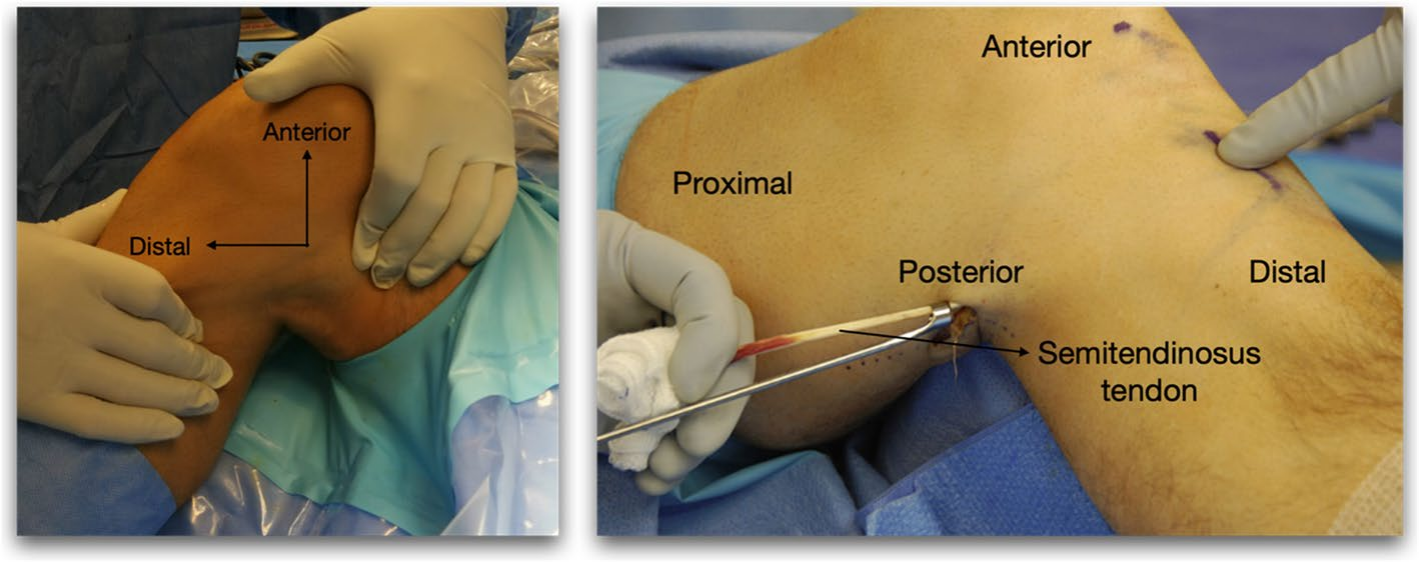
Posterior harvesting technique; Left: Position of the knee during posterior hamstring autograft harvest. Right: Insertion of the tenotomy for distal release of the semitendinosus tendon

In group P, the hamstring graft was harvested via a posterior approach, palpating the tendon in the popliteal fossa with the knee flexed. A 2–3 cm transverse incision was then made at the level of the popliteal fold and the fascia was opened in the direction of the semitendinosus and gracilis tendons. The semitendinosus muscle tendon was then located, isolated and dissected from the gracilis muscle tendon. The possible bands were released immediately afterwards (Fig. 2). Proximal release was performed at the level of the myotendinous junction using an open tenotomy, and the tendon was subsequently disinserted distally using a closed tenotomy. To prepare the graft, the muscle fibres that would have been harvested adhered to the tendon were removed and a tetrafasciculated graft was generated.

Wounds were closed with staples in both groups. During the postoperative period, all patients had their surgical drain removed when it was below 100 cc and were discharged 2–3 days after the surgery. Drainage did not involve an additional incision in either approach.

Patients in both groups followed the same rehabilitation protocol established at the hospital.

### Data collection

Data was collected by means of a systematic review of the electronic health records of each patient and through individual telephone interviews conducted in March 2021. All data were collected after at least 1 year of evolution since surgery.

### Study variables

The main variables were: harvest time measured in minutes (the timer was started from the moment of the skin incision until the tendon was placed on the table), intraoperative complications (premature cutting of the semitendinosus tendon), postoperative neurological complications (allodynia, dysesthesia, hypoesthesia in the donor area and medial region of the knee), hematoma or infection of the surgical wound or septic arthritis, and the patient’s aesthetic satisfaction regarding the scar from the graft harvest wound based on a Likert-type scale (very satisfied/satisfied/somewhat satisfied/dissatisfied).

Other variables of interest were studied as well: range of knee motion, visual analogue scale (VAS) score for pain during activity (0 was “no pain” and 10 was the highest possible pain), atrophy of the thigh operated on as compared to the healthy thigh (measuring the perimeter of the thigh at 10 cm from the upper pole of the patella), rupture of the graft during follow-up and overall satisfaction with the result of the surgery using a Likert-type scale (very satisfied/satisfied/somewhat satisfied/ dissatisfied).

### Statistical analysis

Qualitative variables were described by their frequency and the corresponding percentage. Quantitative variables with a normal distribution were described by their mean and standard deviation, while quantitative variables that did not have a Gaussian distribution were described by their median and interquartile range (IQR).

The groups were compared using the Chi-squared test, Fisher's exact test, Student's T test and Mann–Whitney U test. The software SPSS 25.0 (Chicago, IL) was used and the statistical significance was set to *p* < 0.05.

For the sample size, the G*POWER Software version 3.1.9.7 was used, the calculation of the sample size was performed using the test t of independent samples to compare averages of two groups (A and P). Considering a significance level of 0.05, power of 0.80 and effect size of 0.57 (conservative size), obtaining a sample size of 100 patients (50 in each group).

## Results

### Demographic data

The mean follow-up time was 31 ± 19 months and the sample size was *N* = 100, with 50 patients in each study group (group A: anterior approach; group P: posterior approach). The male/female ratio was 9/1 (A = 44/6 and *P* = 45/5); mean age was 32 ± 13 years (A = 29,86 ± 8,84 and *P* = 32,74 ± 9,46); and football was the most frequent triggering traumatic event, representing 56% of the cases in group A and 56% in group P.

### Harvest time and total operative time (Table 1)

**Table 1:** Tendon harvest time and total operative time; Table representing graft harvest time and total operative time in each group

**Time**			*P* value
	**Group A**	**Group P**	
**Tendon harvest time (minutes)**	9.5 (IQR 7.55–10.40)	5.25 (IQR 4.09–6.3)	0,0005
**Total operative time (minutes)**	68 ± 12,51	63 ± 12,07	0,068

The median harvest time was 9.5 min (IQR 7.55–10.40) in group A. In group P, it was significantly shorter, at 5.25 min (IQR 4.09–6.3) (*p* < 0.05).

However, mean total operative time was 68 ± 12.51 min in group A, and 63 ± 12.07 min in group P, which is not a statistically significant difference (*p* = 0,068).

### Intraoperative complications

There was only one case of premature cutting of the semitendinosus tendon in group A, resulting in a tendon shorter than expected.

### Postoperative complications (Table 2)

**Table 2:** Post-surgical complications; Table representing post-surgical complications: hematoma in the donor area, surgical wound infection or septic arthritis, graft rupture at follow-up and sensory complications in the donor area or medial region of the leg

**Postoperative complications**			***P*** **value**
	**Group A**	**Group P**	
**Hematoma**	2 patients	3 patients	1
**Post-surgical infection**	1 patient	1 patient	0,318
**Graft rupture**	3 patients	1 patient	0,617
**Sensory complications**	8 patients	1 patient	0,031

In group A, 14 patients presented postoperative complications: 16% (8 patients) experienced allodynia, paresthesias or dysesthesia in the graft donor area or in the anterior part of the knee; 4% (2 patients) had hematoma in the harvest wound; in 2% (1 patient) the surgical wound became infected and 6% (3 patients) presented rupture of the graft during follow-up (all related to a new traumatic event). In group P, 6 patients presented postoperative complications: 2% (1 patient) experienced sensory issues similar to the aforementioned; 2% (1 patient) had septic arthritis that was resolved with arthroscopic lavage and graft maintenance; and 2% (1 patient) had rupture of the graft at follow-up (also associated with new trauma).

There were only statistically significant differences (*p* < 0.05) in the variables of neurological complications (allodynia, paresthesias or dysesthesias in the donor area or the medial area of the knee), as presented in Table 2.

### Range of motion

Mean postoperative joint movement was 127.8° ± 6.15° in group A and 129.4° ± 2.4° in group P, without this being a statistically significant difference.

### Circometry

No statistically significant differences were found in terms of atrophy of the operated thigh: a median of 1 cm (IQR 0–2) in group A versus 1 cm (IQR 0–1) in group P.

### VAS

The mean VAS score measured during the patient's usual activity was 0.94 ± 1,9 in group A and 0.8 ± 1,62 in group P. This difference was not statistically significant either.

### Aesthetic satisfaction (Table 3)

**Table 3:** Aesthetic satisfaction; Table representing aesthetic satisfaction of the patient according to a Likert-type scale in each group

**Aesthetic satisfaction**					***P*** **value**
	**Very satisfied**	**Satisfied**	**Little satisfied**	**Dissatisfied**	
**Group A**	80% (40 patients)	16% (8 patients)	4% (2 patients)	0% (0 patient)	0,005
**Gruop P**	92% (46 patients)	8% (4 patients)	0% (4 patients)	0% (0 patient)	

Regarding the patient’s evaluation of the aesthetic result of the surgery, 80% (40 patients) in group A and 92% (46 patients) in group P were very satisfied, 16% (8 patients) in group A and 8% (4 patients) in group P were satisfied and 4% (2 patients) in group A and no patients in group P were somewhat satisfied. In neither of the two groups were there any patients who were dissatisfied.

These differences between groups A and P were statistically significant (*p* < 0.05), as presented in Table 3.

### Overall satisfaction (Table 4)

**Table 4:** Overall satisfaction; Table representing overall satisfaction of the patient according to a Likert-type scale in each group

**Overall satisfaction**					***P*** **value**
	**Very satisfied**	**Satisfied**	**Little satisfied**	**Dissatisfied**	
**Group A**	72% (36 patients)	24% (12 patients)	4% (2 patients)	0% (0 patient)	0,35
**Gruop P**	78% (39 patients)	14% (7 patients)	8% (4 patients)	0% (0 patient)	

Regarding the overall results of the surgery, 72% (36 patients) of patients in group A and 78% (39 patient) in group P were very satisfied, 24% (12 patients) in group A and 14% (7 patients) in group P were satisfied and 4% (2 patients) in group A and 8% (4 patients) in group P were somewhat satisfied.

These differences between groups A and P were not statistically significant (*p* = 0,35), as presented in Table 4.

## Discussion

The most important of this study is that the posterior approach to harvesting the ipsilateral hamstring graft obtained better results than the anterior approach in terms of the patient’s aesthetic satisfaction. This study aimed to assess the potential advantages of the posterior approach in the popliteal fossa for hamstring harvesting during primary ACLR surgery and to compare the results with the classic anteromedial approach.

Only two authors have published studies presenting their positive experience with the aesthetic satisfaction of patients who underwent a posterior approach [10, 19]. In the series published by Kodkani et al. [10], patients on whom a posterior approach was used were less bothered by their scar and all were very satisfied with the resulting aesthetic appearance. After evaluating 90 patients operated on using the posterior approach and an anteromedial mini-incision, Prodromos et al. reported that 80% found the appearance of their knee to be better than that of other patients operated on using an anteromedial approach published in other series [19]. Moreover, 67% of patients were “very satisfied” and the majority gave importance to the appearance of their knee, especially women. However, there are no publications that compare this variable between the two approaches. In this study, the patients who underwent the posterior approach were more satisfied with the aesthetic result of the scar than those who underwent the anteromedial approach, with 92% and 80% being “very satisfied”, respectively. This result is greater than that of Kodkani et al. and Prodromos et al. and may be related to the anterior mini-incision made for the hamstring harvest, which in this study was shorter since the distal disinsertion was performed using the posterior approach with a closed tenotomy. In addition, the good results for aesthetic satisfaction may be due to the fact that the incision is shorter than that made with an anteromedial approach [5], as well as it being located in an anatomical region that in most cases is not within the patient’s line of sight. And because it is in the popliteal fold, it is often indistinguishable. In contrast, among the patients who underwent the posterior approach there were some who reported discomfort from the scar, as well as swelling and itching at various times of day. This variable was not measured since it was not included in the study design but may be due to the scar being located in a flexure exposed to friction and high-frequency sweating.

The anteromedial approach is usually performed through a longitudinal incision over the pes anserum, a structure close to the saphenous nerve and its infrapatellar and sartorial branches. At the knee, the saphenous nerve curves around the sartorius muscle, with its branches passing through the fascia of the sartorius muscle to innervate the overlying skin. It is most likely due to this anatomical layout that the incidence of iatrogenic injury to these nerves is greater when this type of approach is used. Sanders et al. [22] reported injury of this type in 74% of the cases; Papastergiou et al. [18] reported a rate of 39.7%; Franz et al. [5] found a rate of 14%; and Almazán et al. [1] reported a rate of 8.3%. This data highlights the high probability of sensory disturbances when the classic anteromedial approach is employed. In this study, sensory complications occurred in 16% of cases, which aligns with the most recent study conducted by Franz et al. However, the high rate reported by Sanders et al. and Papastergiou et al. could be due to the use of a vertical approach. Both of these studies found a higher incidence of injury to the infrapatellar and/or sartorial branches of the saphenous nerve when using this type of incision as compared to the oblique incision employed in this study due to the anatomical layout of the infrapatellar branch of the saphenous nerve and its terminal branches, which cross perpendicularly to the skin incision made. It is also possible that the methodology used to measure sensory disturbances influenced the results of these studies. Sanders et al. measured sensory disturbances via an anonymous questionnaire, a method that is more sensitive to subjectivity and in which dissatisfaction with the result could have been reflected as a higher rate of sensory problems.

The sartorial branch of the saphenous nerve is superficial to the sartorius fascia, which protects it during the graft harvest via the posteromedial approach [10]. Moreover, five centimeters from where it passes through the subcutaneous cellular tissue, the nerve runs next to the gracilis muscle tendon, which makes iatrogenic injury more likely with the anteromedial approach [22]. When Prodromos et al. [20] described the technique for the posterior harvest approach, they did not report any neurovascular complications. Nor did Dujardin et al. [4] or Franz et al. [5] report any sensory disturbances in their study of patients who underwent the posterior approach. In this study, 2% of patients in the posterior approach group experienced sensory disturbances. This finding may be due to not having performed the approach with the hip in external rotation to relax the infrapatellar branch, or to the inadvertent injury of the protective fascia of the sartorius that protects the nerve, the latter being more unlikely, as already described by Kodkani et al. [10]. Another possibility is that it was a false positive, since the data was collected by phone and the patient may not have correctly understood the type of impairment they were asked about. Furthermore, this data collection method may be more sensitive to subjective complaints, and while this may encourage greater openness from patients, it might also create a selection bias for those patients who are not satisfied, potentially resulting in an artificially higher perceived rate of nerve injury, as described by Sanders [22].

In addition to the lower rate of neurological complications and improved aesthetic appearance, the posteromedial approach was faster. Intertendinous bands between the semitendinosus and the medial gastrocnemius are very common [26]. While these bands can be observed directly and identified in the posterior approach, it is more complicated in the anterior approach [10, 17, 19, 22]. Prodromos et al. [19] showed that it was easier to identify the semitendinosus tendon and release it from its bands using the posteromedial approach. Franz et al. [5] reported a harvest time of 1 min and 21 s with the posterior approach versus 5 min and 15 s with the anterior approach. In this study, harvest time was significantly shorter using the posterior approach, with a mean of 5 min and 25 s as compared to 9 min and 50 s when performing the anterior approach. This variation from the findings published by Franz et al. could be due to a difference in how the time measured, since Franz et al. do not specify when they started and stopped timing graft harvest time, and in the present study the timer was started from the moment of the skin incision until the tendon was placed on the table. In addition, only one tendon was removed when using this approach. When the anterior approach was used, both the semitendinosus and gracilis tendons were usually removed. Nevertheless, it must be taken into account that identifying the semitendinosus tendon and differentiating it from the gracilis tendon and the medial collateral ligament takes longer than extracting both tendons given their convergence at the distal level.

No statistically significant differences were found that can be attributed to the approach as regards the pain visual analog scale score, the presence of hematoma or infection of the surgical wound, joint movement, atrophy of the thigh operated on compared to the contralateral thigh, or overall patient satisfaction with the surgery result.

It is worth noting that the posterior approach also poses some disadvantages. An additional incision at the anterior level is required to make the tibial tunnel and grafts harvested are shorter [5]. However, although the grafts harvested could be shorter, their functional result is good; Streich et al. [25] showed that a tetrafasciculate graft of the semitendinosus tendon achieved good clinical results at 10 years follow-up. Nowadays, the use of retrograde drills to make tunnels and “all-inside” techniques have made it possible to reduce the additional anterior incision required when harvesting the graft through the popliteal fossa [2].

As for limitations, it should be noted that this is a non-randomized retrospective observational study and, therefore, may potentially have associated biases. However, both groups had similar demographic characteristics and only differed in the approach used to harvest the hamstring graft, thus making them comparable. In addition, although the sample size was not previously defined, this study could reveal cause-effect relationships such as those previously mentioned since it is the same as that in the Franz et al. study [5], which described findings which some of ours align with. Furthermore, the results match those reported in the most recent literature by Kodkani, Prodromos, Franz and Dujardin [4, 5, 10, 19]. Despite the fact that the male/female ratio was 9/1, differences were found in terms of aesthetic satisfaction. If the number of women in the series were increased, the differences may be increased given the greater aesthetic demand by female patients, as already reflected in the series by Prodromos et al. [19]. Another limitation was data collection by phone, which puts the data at risk of patient subjectivity, such as their determination of the type of sensory disturbance in the anterior aspect of the knee. Another limitation was the fact that they were two different surgeons, which could lead to significant differences in the development of the technique. However, they were surgeons specialised in this technique and harvesting approach.

## Conclusions

In this study, the posterior approach to harvesting the ipsilateral hamstring graft obtained better results than the anterior approach in terms of the patient’s aesthetic satisfaction, lower rate of neurological complications (allodynia, paresthesias and hypoesthesia in the anterior region of the knee and the leg) and shorter hamstring harvest time. Furthermore, there were no differences in VAS scores, joint movement, atrophy of the operated thigh compared to the contralateral thigh, or overall patient satisfaction.
